# Ovarian seromucinous carcinoma: an independent epithelial ovarian cancer?

**DOI:** 10.1186/s13048-023-01100-w

**Published:** 2023-01-20

**Authors:** Yi Hu, Kun Fu, Huining Liu, Qiongqiong He, Xiaoqin Qiu, Wenqing Yang, Yu Zhang

**Affiliations:** 1 Department of Gynecology, Xiangya Hospital, Central South University, Changsha, Hunan China; 2 Department of Pathology, Xiangya Hospital, Central South University, Hunan Changsha, China; 3 Department of Pathology, School of Basic Medicine, Central South University, Changsha, Hunan China; 4 Gynecological Oncology Research and Engineering Center of Hunan Province, 86 Xiangya Road, Changsha, Hunan 410018 China

**Keywords:** Ovarian seromucinous carcinoma, Epithelial ovarian carcinomas, Retrospective study, Clinicopathological features, Prognostic evaluation

## Abstract

**Background:**

2020 World Health Organization Classification of Female Genital Tumors removed ovarian seromucinous carcinoma as a distinct entity and recategorized it as ovarian endometrioid carcinoma with mucinous differentiation according to its pathological features. The aim of this study was to find whether ovarian seromucinous carcinoma truly represented a distinct category of ovarian tumors or an analogue of ovarian endometrioid carcinoma.

**Methods:**

Twelve patients diagnosed with ovarian seromucinous carcinoma and received surgery at the Xiangya Hospital from January 2010 to December 2019 were included in this study. Clinicopathological features such as clinical symptoms, serological indicators, surgical information, postoperative findings, chemotherapy sensitivity, follow-up information, HE staining and IHC staining images and other clinicopathologic features were collected. Using t-test and Kaplan Meier to perform statistical analysis. Pathological review was conducted using the 2014 World Health Organization criteria. All pathological diagnoses were reviewed by two experienced pathologists.

**Results:**

The age of 12 patients diagnosed with ovarian seromucinous carcinoma ranged from 23 to 68 years, with a median age of 46.8 years. Serum level of CA125 was elevated in 10 patients, and CA125/CEA ratio was less than 25 in 6 patients. Eleven patients underwent radical ovarian cancer surgery, and one patient underwent fertility preservation surgery. The progression free survival and overall survival of ovarian seromucinous carcinoma is 46.8 months and 50.2 months. Kaplan-Meier survival curve showed that the prognosis of ovarian seromucinous carcinoma and ovarian endometrioid carcinoma was significantly different (*P* = 0.03). The prognosis of ovarian seromucinous carcinoma and ovarian mucinous carcinoma was similar.

**Conclusion:**

Although ovarian seromucinous carcinoma and ovarian endometrioid carcinoma are similar in pathologic morphology, their clinical features and prognosis are significantly different. The signs, serum biomarker and prognosis of the ovarian seromucinous carcinoma are similar with ovarian mucinous carcinoma. Therefore, ovarian seromucinous carcinoma is not suitable to be directly classified as ovarian endometrioid carcinoma.

## Background

Epithelial ovarian carcinomas (EOCs) are primarily classified into serous, mucinous, clear cell, endometrioid, transitional, and squamous cell carcinoma [1]. Seromucinous ovarian tumors are rare neoplasms formerly classified with mucinous tumors, as the Müllerian or endocervical subtype. As early as in 1976, Fox and Langley firstly introduced the seromucinous tumor to describe a tumor composed of endocervical type mucinous epithelium and serous-type cells [2]. Then in 2014, World Health Organization (WHO) firstly introduced the ovarian seromucinous tumors classification as a distinct pathologic type of ovarian epithelial tumors [3].

Ovarian seromucinous carcinoma (OSMC), as a kind of malignant seromucinous ovarian tumor, has aroused widespread controversy in the field of pathology in recent years. Some pathologists didn’t consider the OSMC can be a well-defined histological entity. Because its morphological and immune-phenotype overlapped with other types of ovarian tumors, especially ovarian endometrioid carcinoma (OEC) and low-grade serous carcinoma (LGSOC) with mucinous differentiation [4]. Therefore, the 5th edition of WHO Classification of Female Genital Tumors published in 2020 has removed OSMC as a distinct entity and now considers it as a subtype of OEC [5]. But due to its rarity, there is no clinical and pathological information about OSMC and clinical study to compare OSMC and other mainly EOCs clinicopathological features has been reported.

To provide additional knowledge to this controversy, here we present 12 cases of OSMCs diagnosed and treated in our hospital from 2010 to 2019. Based on a retrospective review of these cases and current literature, we attempt to enhance the understanding of this rare but serious disease.

## Materials and methods

### Study population

A total of 106 female patients were pathological diagnosed of different EOCs who received surgery at the Xiangya Hospital from January 2010 to December 2019. Among this cohort, 12 cases of OSMC were diagnosed by two experienced pathologists based on the 2014 WHO classification. Twenty-four cases of OEC, ovarian serous carcinoma (OSC) and ovarian mucinous carcinoma (OMC) and 22 cases of clear cell carcinoma (OCCC) diagnosed from 2010 to 2019 in Xiangya Hospital were included in this study as paired cohort. To eliminate the error, we also collected all the 27 cases of OEC and 36 cases of OMC from 2010 to 2019 in Xiangya Hospital. Besides that, 52 cases of OSC were collected in 2015. These 149 patients were included in the whole cohort. Clinicopathological features such as clinical symptoms, serological indicators, chemotherapy sensitivity, microscopic manifestations and prognosis were collected through the electronic medical records. Follow-up ended in December 2020. This study was approved by the Clinical Research Ethics Committee of the Xiangya Hospital (No. 2017068222).

### Histopathological evaluation and immunohistochemical analysis

Pathological review and re-diagnosis were conducted for all cases by 2 observers according to 2014 WHO criteria [3]. For IHC staining, we observed progesterone receptor (PR), estrogen receptor (ER), Carbohydrate antigen 125(CA125), cytokeratin Pan (CK-Pan) and PAX-8.

### Statistical analysis

Statistical analysis was performed with the SPSS statistical software package. Using t-test to compare differences between different EOCs. Survival probability was estimated by Kaplan Meier analysis with log-rank product limit estimation. For all analyses, statistical significance was indicated at a *p* value of<0.05.

## Results

### Clinical manifestations and treatment of OSMCs patients

Details of clinicopathological features from patients diagnosed as OSMCs are shown in Tables 1 and 2. Ages of 12 patients of OSMCs ranged from 23 to 68 years with mean and median age of 46.8 and 47.5 years respectively. Two patients (16.7%) had bilateral pelvic masses and five (41.7%) had unilateral pelvic masses. Ascites were detectable in 10 cases and cancer cells were found in ascites in 3 cases.8 cases (66.7%) of OSMC visited doctors due to the pelvic masses. Ten cases are in stage I-II and 2 cases are in stage III according to the 2018 FIGO classification. One patient retained reproductive function and others received Radical operation.11 cases received Platinum-based chemotherapy.

**Table 1:** Clinicopathologic features of the women with a diagnosis of OSMC from 2010 to 2019

ID	Age (years)	Signs	Location	Ascites	FIGO Stage	Grade
1	47	pelvic lump	Left	N^a^	IA	None
2	53	pelvic lump	Bilateral	N	IB	2
3	60	abdominal distention	Right	Y	IC	2
4	66	abnormal uterine bleeding	Left	Y	IA	N
5	49	pelvic lump	Right	Y	IA	1
6	32	pelvic lump	Left	Y	IIA	N
7	45	pelvic lump	Left	Y	IC	3
8	26	pelvic lump	Right	Y	IIIA	2
9	46	pelvic lump	Bilateral	Y	IIIC	2
10	68	abdominal distention	Right	Y	IIB	3
11	48	abdominal distention	Right	Y	IA	N
12	23	pelvic lump	Left	Y	IIB	1

^a^Y means the patient had ascites samples; N means the patient did not have ascites samples

**Table 2:** Management and prognosis of patients with the OSMC from 2010 to 2019

IDX	Surgery	Chemotherapy	PFS (months)	OS (months)
1	TAH + BSO + OM	None^b^	NA^c^	NA
2	TAH + BSO + OM	TP	96	96
3	TAH + BSO + OM	TO	91	91
4	TAH + BSO + OM	TO	88.5	88.5
5	TAH + BSO + OM	TC	78	78
6	TAH + BSO + OM + PEN	TC	10	11
7	TAH + BSO + OM	TP + IC + Doxorubicin	2.5	36
8	TAH + BSO + OM + PEN+PAN	TP	16	18
9	TAH + BSO + OM + PEN+PAN	TC	22	28
10	TAH + BSO + OM	None	NA	NA
11	TAH + BSO + OM + PEN+PAN	TP	38	38
12	LSO + OM + PAN^a^	TP	17.5	17.5

*ATH* abdominal total hysterectomy, *BSO* bilateral salpingo-oophorectomy, *LSO* left salpingo-oophorectomy, *OM* omentectomy, *PEN* pelvic lymph node dissection, *PAN* para-aortic lymph node dissection, *TC* paclitaxel and carboplatin, *DP* docetaxel and carboplatin/ Cisplatin, *DO* docetaxel and oxaliplatin, *TO* paclitaxel and oxaliplatin, *IC* irinotecan and carboplatin

^a^ Fertility preserving surgery

^b^ No chemotherapy

^c^ loss to follow-up

Clinicopathological features of all EOCs cases are shown in Table 3. OEC, OSC, OMC were paired in a two-fold ratio with OSMC according to age and stage. There were only 22 cases of OCCC had been diagnosed from 2010 to 2019 in Xiangya Hospital, so all of OCCCs were included. There was no significant difference in accompanied by endometriosis, residual lesions and chemotherapy among all types of EOC. There is a striking difference in CA125/ CEA ratio. In OSMC, the ratio of CA125/ CEA is less than 25 in 6 cases (50%), while OEC is 9% (*p*<0.05) and OSC is 33.3%, but OMC is 58%.

**Table 3:** Compare the OSMC with other EOCs diagnosed from 2010 to 2019

	Ovarian seromucinous Carcinoma (*n* = 12)	Ovarian endometrioid carcinoma (*n* = 24)	Ovarian serous carcinoma (*n* = 24)	Ovarian mucinous carcinoma (*n* = 24)	Ovarian Clear Cell Carcinoma (*n* = 22) **
**Age (years)**	46.83 ± 11.04 (23-68)	46.83 ± 8.93 (24-63)	47.33 ± 13.15 (21-69)	46.88 ± 12.97 (24-70)	51.5 ± 6.39 (39-67)
**Biomaker**					
CA125(U/ml)					
<35	2	2	6	9	7
>35	10	22	18	15	15
CEA (ng/ml)					
<5	9	23	20	18	17^***^
>5	3	1	4	6	1
CA125/CEA					
>25	6	22^*^	16^*^	10	10
≤25	6	2	8	14	8
**Ascites**					
Yes	10	19	15	14	15
No	2	5	9	10	7
**FIGO stage**					
I-II	10	19	20	18	15
III-IV	2	5	4	6	7
**Endometriosis**					
Yes	0	3	0	2	4
No	12	21	24	22	18
**Residual lesions**					
<R1	11	23	22	20	20
>R1	0	1	2	4	2
None	1	0	0	0	0
**Response to Chemotherapy**					
Sensitivity	10	19	17	15	15
Resistance	1	0	4	4	3
None	1	5	3	5	4
**PFS (month)**	46.8	42.8	40	40	29.8
**OS (month)**	50.2	46	46.4	42	31.4

*Means the results were significantly different from those of OSMC (*p*<0.05), ** Twenty-two cases were diagnosed in Xiangya Hospital from 2010 to 2019; ***The 4 patients’ results of CEA were missing

### Prognostic analysis

By the December 2020, in paired cohort, survival data were available for 90 cases, as 16 patients were lost to follow up. In patients lost to follow-up, 2 cases were OSMC, 5 cases were both OEC and OMC, 4 cases were OCCC. The PFS and OS for different ovarian carcinoma is showed in Table 3.

**Fig. 1:**
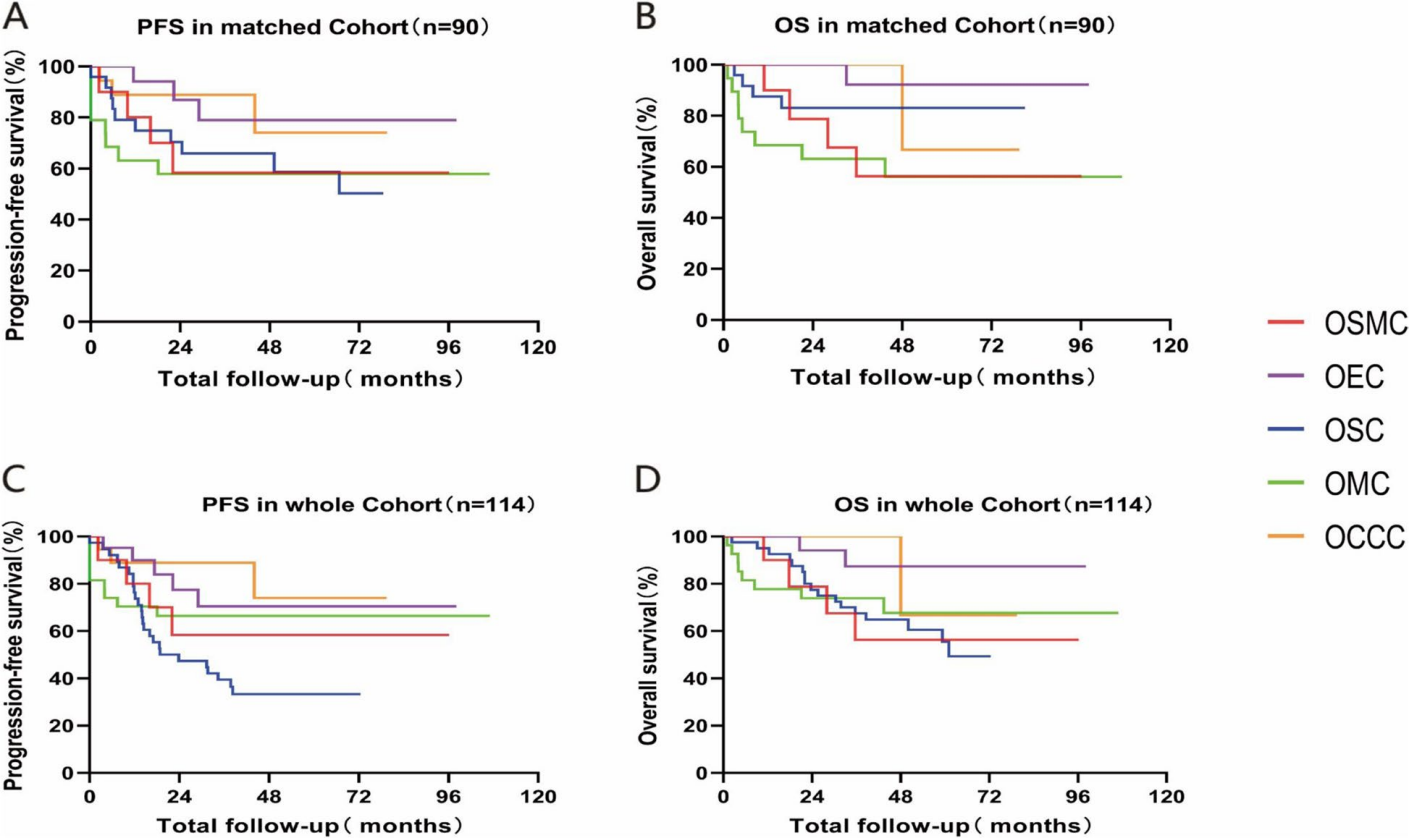
Kaplan-Meier estimates for time to follow-up. **A**. PFS of OSMC comparing with the other EOCs (matched cohort) during 2010 to 2019; **B**. OS of OSMC comparing with the other EOCs (matched cohort) during 2010 to 2019, the *p* = 0.03 refer to OSMC vs OEC; **C**. PFS of OSMC comparing with the other EOCs during 2010 to 2019(All the cases of OSC were diagnosed in 2015); D. OS of OSMC comparing with the other EOCs during 2010 to 2019

Overall survival was dependent on the stage of the disease (Fig. 1A and B). By the end of 2020, patients diagnosed at stage I had a survival of almost 85%, at stage II 60%, at stage III 55%; Similar findings were also found for progression-free survival. As for histological subtype, patients with OSMC and OMC showed a 5-year survival of less than 60%, OEC showed a 5-year survival of almost 90% and OSC a 5-year survival of almost 80%. The difference in survival were statistically significant in OSMC and OEC (*p* = 0.03). Results were similar for progression free survival.

Because the number of OSMC is relatively small, to eliminate the error, this study compared OSMC with all the OEC, OMC and OCCC between January 2010 and December 2019 and all the OSC in 2015, survival data were available for 114 cases (Fig. 1C and D). Patients with OSMC, OSC, OMC and OCCC showed a 5-year survival of less than 70% while OEC showed a 5-year survival of almost 90%.

Although there is no significant difference in survival between different types of EOCs, it also can be seen that the prognosis of OEC is better than other EOCs.

### Microscopic features in OSMCs

**Fig. 2:**
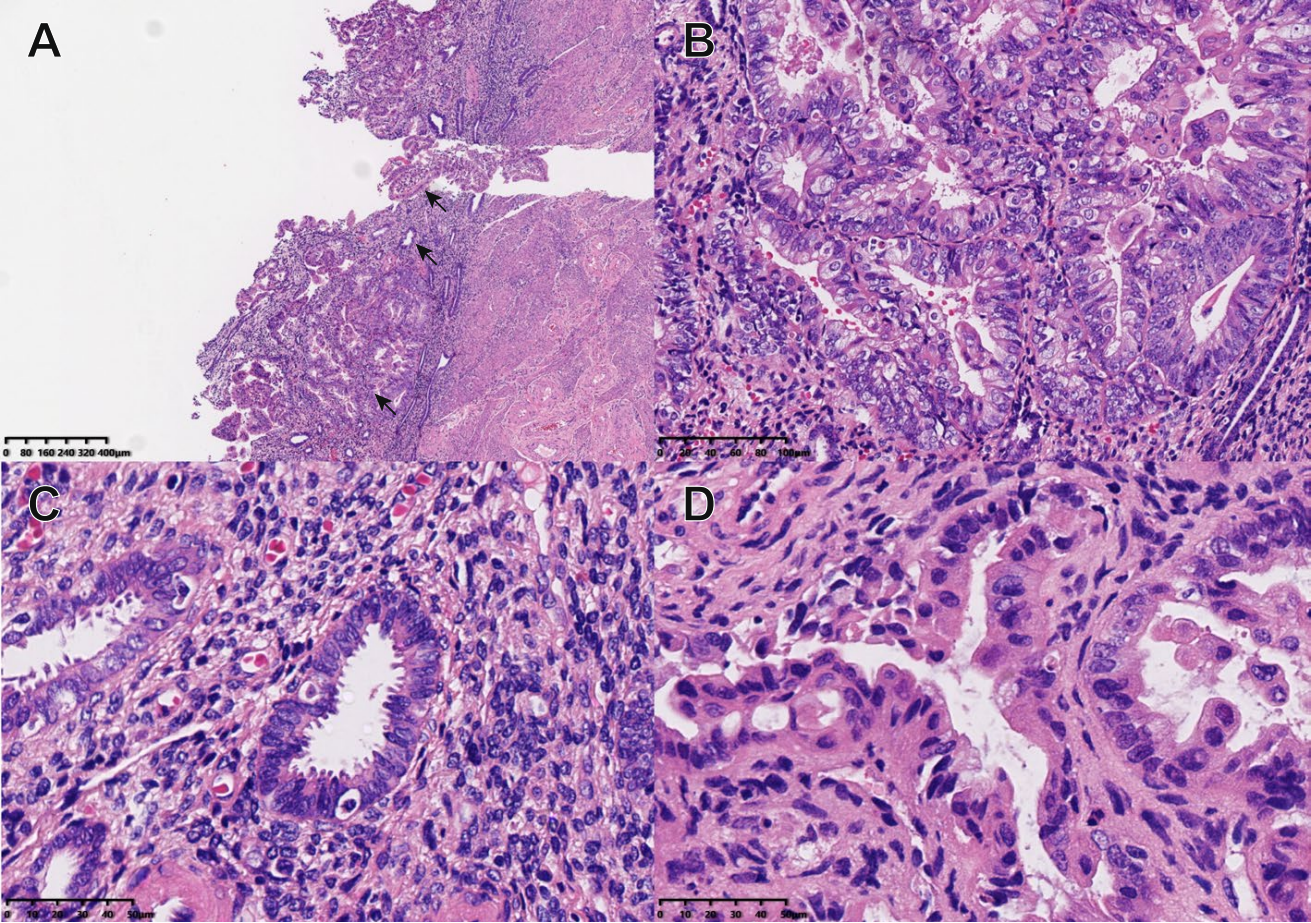
Characteristic pathological images of OSMC with HE staining. **A**. Pathological images of OSMC (HES × 40). **B**. The OSMC contains cervical tubular mucous epithelium (HE× 400). **C**. Small nipples (HES × 400). D: Squamous metaplasia (HES × 400)

OSMC has its own unique pathological characteristics (Fig. 2A). Architecture was papillary in most cases, characterized by large firbo-oedematous papillae. At the same time OSMC has a mucous epithelium in this model of the cervical canal (Fig. 2B) and show a small nipple (Fig. 2C), while some of cases can present Squamous metaplasia (Fig. 2D).

**Fig. 3:**
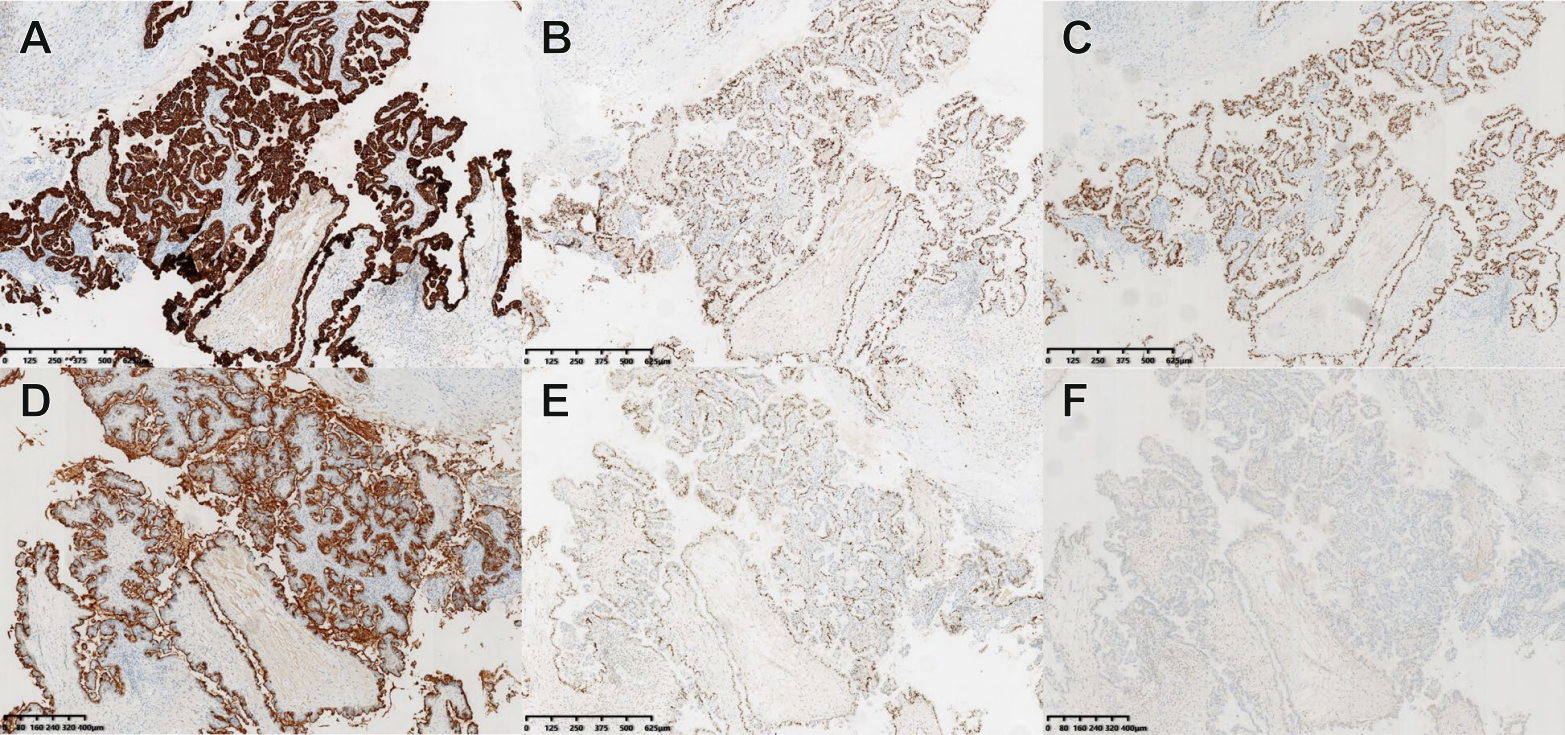
Characteristic pathological images of OSMC with IHC staining. In IHC staining, OSMC mainly expressed Mullerian markers and WT1 is often not expressed. **A**. CK-Pan (+). **B**. ER (+). **C**. PAX-8(+). **D**. CA125(+). **E**. PR (+). **F**.WT1 (−)

Immunohistochemical was performed in Fig. 3. OSMC mainly expressed Mullerian type markers such as Cytokeratin Pan (CK-Pan), Estrogen Receptor (ER), Progesterone Receptor (PR) and Pairing box gene 8 antigen (PAX-8). ER and PR often express in ovarian carcinoma. CK-Pan is used to identify epithelial and non-epithelial components. Pax-8 is specific expression in ovarian cancer. Our OSMC expressed IHC stains for CK-Pan (Fig. 3A), ER (Fig. 3B), PAX8 (Fig. 3C), carbohydrate antigen 12(CA125) (Fig. 3D), PR (Fig. 3E) and was negative for Wilms tumor protein (WT1) (Fig. 3F). WT1 usually express in ovarian cancer especially in High-grade serous carcinoma. In OCCC and OEC, WT1 tends to be negative.

## Discussion

The 2014 WHO Classification of Tumors of the Female Reproductive Organs introduced a new category of ovarian neoplasm designated seromucinous tumors [3]. The recognition of this distinctive group is an important addition to the classification of epithelial ovarian tumors. To the best of our knowledge, in 1976 when Fox and Langley introduced the term seromucinous tumor to describe a tumor composed of endocervical type mucinous epithelium and serous-type cells [2]. Then in 1988 Rutgers and Scully divided similar appearing borderline tumors into two categories. One was composed of pure endocervical-type epithelium and another one was composed of a mixture of endocervical-type mucinous, serous, endometrioid and indifferent cells with abundant eosinophilic cytoplasm [6, 7]. Later, in 1993 Hendrickson and Kempson resurrected the term “seromucinous tumor” that a term that Shappell adopted in 2002 [8]. The previous studies were almost described the OSMCs from the pathologic aspects, and our study is firstly comparing OSMC with other EOCs to prove that OSMC is different with OEC from clinical perspective.

OSMC has similar clinical characteristics to OMC. Most OSMCs, like OMC, appear as large lumps, so a majority of OSMC are stage I or II. In our series, the prognosis of OSMC is correlated with stages. In general, patients at earlier stage have a better prognosis except case 7. The PFS of this case in OSMC is 2.5 months. One possible reason is that the grade of the lesion is three. This may be the reason of its resistance to chemotherapy. The patient received multiple chemotherapy regimens, but still died in December 26, 2017. As for biomarker, 50% cases’ ratio of CA125 to CEA of OSMC is less than 25 in OSMC, while OEC is 9% and OSC is 33.3%, but OMC is 58%. In ovarian cancer, about 5-15% of all ovarian malignancies are metastases from another malignancy. The majority of these metastases have a gastrointestinal origin, but also metastases from breast, skin or other gynaecological origin occur [9]. The ratio of CA125 and CEA differentiates better between EOC and ovarian metastases from gastrointestinal neoplasms than one of these markers alone [10]. A previous study also showed a sensitivity of 73% and specificity of 63% when a cut-off value for the CA125/CEA ratio of 25 was used compared to a sensitivity of 78% and specificity of 50% for CA125 alone to distinguish EOC and gastrointestinal neoplasms and a cut-off value of 25 has demonstrated high accuracy, and thus it has been used in clinical practice [11, 12]. OMC usually performs he biological characteristics of gastrointestinal neoplasms, so we often use the ratio of CA125 and CEA to distinguish OMC and other EOCs. In our study, more than 50% cases’ ratio of CA125 to CEA is less than 25 both in OSMC and OMC. Another point is that OMC is usually very large primary tumors that generate symptoms while the disease is still localized to the ovary [13]. And in 12 OMSC cases, about 66.7% cases are because of a pelvic mass. These all may suggest that OSMC has some biological characteristics of OMC.

In matched cohort the prognosis of OSMC is probably closer to that of OMC and different with OEC(*p* = 0.03). The prognosis for OEC is better than other EOCs. A potential explanation is that most OEC diagnosed at an early stage. In matched cohort, the prognosis for OSC is better than OSMC, but when change the OSC patients in 2015, the prognosis for OSC become worse. One possible reason is that most cases of the OSC in the matched cohort are early stage and lower grade, but High-grade serous carcinoma was diagnosed more often in higher stage in a natural year, and this did affect prognosis. In the whole cohort, the prognosis was not significantly related to the type of EOCs.

Histologically, our results show that firbo-oedematous papillae, squamous metaplasia, mucous epithelium in this model of the cervical canal and small nipples are the unique pathological characteristics of OSMCs. On the previous studies, the background of endometriosis was found in 36% of the cases, with ectopic endometrium and serous carcinoma transition visible in some tumors. But in our study, endometriosis was not found in any of the cases, even if we focused on past medical history of patients, we cannot find endometriosis in one case.

As for the immunophenotype of OSMC, the phenotype of gastrointestinal and mucinous tumors is quite different from that of OSMC [14]. Gastrointestinal and mucinous tumors often express markers of gastrointestinal differentiation: CK20 and CDX2, while OSMC expressed the markers of Mullerian epithelium: ER, PR, CA125, mesothelin. Moreover, PAX-8 was strongly expressed in OSMC, which was different from mucinous tumors but similar to serous tumors. In our study, OSMC also expressed the markers of Mullerian epithelium and PAX-8 was strongly expressed.

At present, there are few studies on the molecular characteristics of OSMC. The ARID1A gene is reported to be mutated in half of clear cell ovarian cancers and 30% of endometrioid cancers [15, 16]. In a study of 32 patients with OSC, the mutations included KRAS, PIK3CA, PTEN, and ARID1A [17]. And a study in 2020 demonstrated a distinct mutational landscape of OSMC in which (1) KRAS is invariably mutated, (2) PIK3CA is frequently mutated, and (3) TERT promoter mutations and DNA mismatch repair deficiencies are absent [4]. So from a molecular point of view, OSMC is probably not belong to OEC and its molecular signature is more like OSC. Because of the time span of our cases, molecular testing was not performed.

## Conclusion

To conclude, in our series, although OSMC and OEC are similar in pathologic morphology, their clinical features and prognosis are significantly different, and some clinical features and prognosis are similar between OSMC and OMC. If independently confirmed, our findings may have significant implications for disease classification and clinical treatment. Of course, only 12 cases were included in this study, this can lead to errors in the results and further large-scale studies examining this in detail are needed.

## Data Availability

Data and materials of this work are available from the corresponding author on request.

## Abbreviations

WHO: World Health Organization
FIGO: International Federation of Gynecology and Obstetrics
EOC: Epithelial ovarian carcinoma
OSMC: Ovarian seromucinous carcinoma
OEC: Ovarian endometrioid carcinoma
OSC: Ovarian serous carcinomas
OMC: Ovarian mucous carcinoma
OCCC: Ovarian Clear Cell Carcinoma
CK-Pan: Cytokeratin Pan
ER: Estrogen Receptor
PR: Progesterone Receptor
WT1: Wilms’ Tumor1
CA125: Carbohydrate antigen 125
